# The Next Opportunity in Anti-Malaria Drug Discovery: The Liver Stage

**DOI:** 10.1371/journal.ppat.1002178

**Published:** 2011-09-22

**Authors:** Emily R. Derbyshire, Maria M. Mota, Jon Clardy

**Affiliations:** 1 Deparment of Biological Chemistry and Molecular Pharmacology, Harvard Medical School, Boston, Massachusetts, United States of America; 2 Unidade de Malária, Instituto de Medicina Molecular, Faculdade de Medicina Universidade de Lisboa, Lisboa, Portugal; The Fox Chase Cancer Center, United States of America

Malaria afflicts 350–500 million people annually, and this debilitating and deadly infectious disease exacts a heavy toll on susceptible populations around the globe. Efforts to find effective, safe, and low-cost drugs for malaria have sharply increased in recent years. Almost all of these efforts have focused on the cyclic blood stage of the disease, partly because the parasites can be easily maintained in culture through addition of human red blood cells to the growth medium, and partly because blood stage infection causes malaria's characteristic symptoms. However, the asymptomatic liver stage, which the parasite goes through only once in its life history, presents the best opportunity for developing drugs that both hit new targets and also could be used in highly desirable eradication campaigns. Recent research, especially on the frequency of differentially expressed genes in blood and liver stage parasites, supports the feasibility of discovering stage-specific drugs. Discovering these drugs will require a high-throughput liver stage phenotypic screen comparable to the existing blood stage screens, and the basic tools for such a screen have recently been created.

## Background

Humans have suffered from the burden of malarial infections for thousands of years, and the disease has greatly influenced human evolution and history [1]–[3]. Malaria remains a devastating disease, and in developing countries within Africa, South America, and Asia, the size of its burden has stifled economic growth and development [2]. Despite successful eradication campaigns in North America and Europe, global cases of the disease show little decline, and current improvements rely on pyrethroid treated bed nets and combination therapeutics containing artemisinin derivatives, both of which are susceptible to emerging resistance [4]. Our ability to counter these vulnerabilities with new agents is hampered by the modest number of fully validated drug targets and our limited understanding of many aspects of parasite biology.

**Figure 1:**
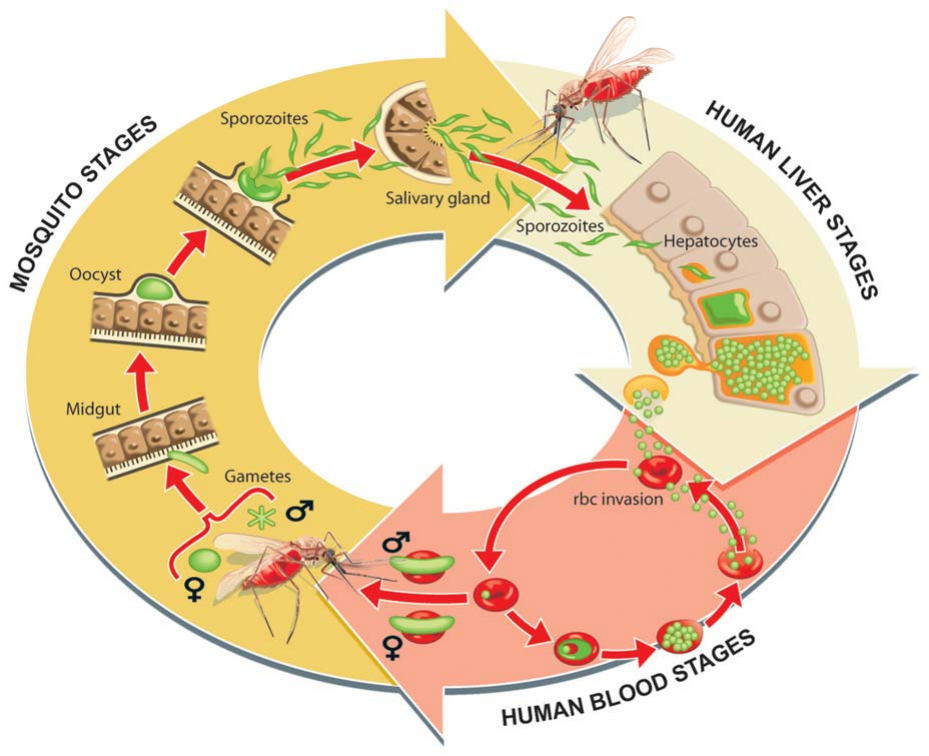
Parasite life cycle in the human host and mosquito vector. Sporozoites that are found in a mosquito's salivary gland are injected into the skin during the blood meal. The sporozoites that reach a blood vessel travel to the liver and traverse several cells before developing in a hepatocyte. Here the parasite numbers grow significantly and they develop into a form that can invade red blood cells to initiate the cyclic asexual stage. During this time some sexual gametocytes also develop and it is this form of the parasite that is taken up by a mosquito. The parasite invades the mosquito's midgut and develops into sporozoites that can infect a new human host. Graphic prepared by Ian Moores Graphics.

Today we understand that malaria is a parasitic disease spread to humans through the bite of an *Anopheles* mosquito. During their obligatory blood meals, infected female mosquitoes transmit protozoan parasites belonging to the genus *Plasmodium*, and their proliferation in the human host causes malaria's symptoms (Figure 1). Parasites from infected humans are transmitted back to the mosquito host in subsequent blood meals. The parasite has a complicated life history. In the mosquito gut, the parasites taken up from the human host differentiate into male or female gametes and produce a motile form that migrates through the mosquito gut wall and transforms into an oocyst that in approximately 2 weeks produces and releases thousands of sporozoites that invade the mosquito's salivary glands. Mosquitoes inject sporozoites into the human host during their blood meals, and the sporozoites travel from the skin, through the blood stream, to the liver. Once in the liver, a sporozoite migrates through several liver cells before it settles, propagates, and produces thousands of merozoites (reviewed in [5]) (Figure 1). This exoerythrocytic phase produces no host symptoms. But once the merozoites infect red blood cells, the symptomatic cyclical erythrocytic stage begins. Its waves of bursting red blood cells and the invasion of fresh red blood cells by the newly released parasites produce malaria's characteristic symptoms. In total, the parasite's complicated life history involves two hosts (mosquito and human), three major life-stages (vector stage, liver stage, and blood stage), and multiple forms. The human stages entail vastly different numbers of parasites as an initial infection can begin with as few as 10 parasites from the mosquito, followed by an expansion by a factor of a thousand in the liver, and ultimately an expansion to several trillion in a mature blood stage infection [5].

*P. falciparum* is the deadliest of the species of *Plasmodium* that infect humans, and it accounts for the majority of malaria infections and virtually all of the malaria-related mortality worldwide [6]. *P. vivax*, *P. falciparum*'s less deadly relative, contributes substantially to malaria's morbidity if not mortality [7], [8]. *P. vivax* and *P. ovale* can hide out in the liver for prolonged periods as hypnozoites, the latent hepatic stage, which can cause relapsing malaria months or even years after the initial infection [9]. Only primaquine clears both liver stage parasites and latent stage hypnozoites, while others target only the liver stage parasites (atovaquone). Parasites can rapidly develop drug resistance but at unpredictable and varying rates. There are global concerns that the efficacy of all currently used anti-malarial drugs will erode, thereby creating a pressing need to develop inexpensive yet effective agents that can both treat and eradicate malaria. Ironically, while current drug development focuses on the well-studied blood stage, the vector and liver stages present better options for an eradication campaign, as targeting these stages will prevent infection and disease.

## The Past and Present of Anti-Malaria Drug Discovery

Several drugs are used in various combinations to treat malaria, but chloroquine and arteminsinin are arguably the most abundantly used agents (reviewed in [10]). Artemisinin comes from a plant (*Artemisia annua*) used in traditional Chinese medicine to treat fevers, and the compound and its derivatives are presently used worldwide in anti-malarial combination therapies (reviewed in [11]). Chloroquine was first synthesized early in the last century, although its history can be traced back to quinine from the bark of cinchona trees, the new world's ethnobotanical contribution to the treatment of malaria. Both artemisinin and chloroquine are effective against the parasite's blood stage. Chloroquine targets heme polymerization in the parasite's food vacuole, while artemisinin's target is not known with any certainty [12]. Neither arteminsinin nor chloroquine is effective against the liver stage in vivo. Drug-resistant *Plasmodium* strains have become prevalent for the currently used blood-stage anti-malarials (reviewed in [13]) and reports of decreased sensitivity towards artensunate in Cambodia (reviewed in [11]) are particularly worrisome.

The high risk of parasite resistance to current therapies highlights the need for replacements, and the most effective replacements are likely to have new targets and new chemotypes to which the parasite has not yet developed resistance. The search for these targets has been frustrated by several factors. Unlike other systems, the sequencing of *Plasmodium* genomes provides few clues about essential genes and/or processes as ∼50% of the genome contains open reading frames of unknown function [14]. Determining gene function has been hampered by the limited tools available to carry out genetic manipulations on the parasite. And to complete the list of complicating factors, parasite proteins are unusually difficult to express and characterize.

In spite of these obstacles, current approaches to discovering drugs with new targets and chemotypes have had some successes, which have come largely from high-throughput screening of libraries of structurally diverse small molecules. Drug discovery screens can be either target-based or phenotypic. Target-based screens are often operationally simpler as they can use pure proteins in a biochemical assay, but they require selecting the target prior to screening, and they will only discover candidate molecules that modulate the chosen target. Several target-based screens for suitable therapeutic agents have been developed in the past 10 years. Histone deacetylases (HDAC) [15], [16], dihydroorotate dehydrogenase (DHODH) [17], [18], dihydrofolate reductase (DHFR) [19], heat shock protein 90 (Hsp90), and enzymes involved in fatty acid biosynthesis [20], [21] have been among the most promising. While these assays have provided compounds that inhibit malaria growth, the lack of species specificity between the *Plasmodium* and human enzymes has limited drug development.

Phenotypic screens involving whole cells or even whole organisms have the obvious advantage of being able to find new targets. But they have the drawback of being more, often much more, complicated to run. The only widely used phenotypic malaria screens involve blood stage parasites and a growth/no growth phenotype. There are several published screens that use varying reporters, but they all exploit the ability of the parasite to be grown in red blood cell culture. The first published high-throughput assay utilized a fluorescent DNA dye to quantify the parasite's growth, or lack thereof [22], and most recently GlaxoSmithKline reported the results of a high-throughput screen measuring the parasite's lactate dehydrogenase (LDR) activity [23]. In addition to these methods, luciferase transgenic parasites [24], RNA probes [25], antibody, and PCR-based [26] detection methods have all been developed and have the potential to be optimized toward the goal of anti-malarial drug discovery. Phenotypic screens typically do not reveal an active molecule's mechanism, and linking a molecule to its target can be very difficult [27]. Target identification for phenotypic blood stage screens has repeatedly identified already known targets, like cytochrome bc1 [23]. In this regard, a recent blood stage phenotypic screen by Novartis that identified a gene important for protein synthesis, a P-type cation-transporter ATPase4, is especially noteworthy [28]. In spite of this and hopefully similar successes in the future, we believe that the greatest opportunity for anti-malarial drug discovery lies in the development of non-blood stage parasite screens.

## The Need for Anti-Malarial Drugs Targeting the Liver Stage

Liver stage drugs have the potential to hit new targets that are not present or essential during the blood stages of malaria, and these drugs would enjoy a tactical advantage over blood stage drugs because many fewer parasites are involved, which should delay the development of resistance [12]. Liver stage drugs may also have activity against the hypnozoites of *P. vivax*, like primaquine, and thereby prevent relapsing malaria. Despite their apparent advantages, progress in developing liver stage drugs has been very limited, and this lack of success undoubtedly reflects the way drugs are currently discovered: large numbers of molecules are screened for blood stage activity and only selected active candidates are screened in the liver stage [12]. Primaquine, anti-folates, and atovaquone are currently used drugs with liver stage activity, but among these only primaquine also has anti-hypnozoite activity.

Primaquine, a distant chemical relative of chloroquine whose target has yet to be determined, can inhibit both liver stage infections and blood stage parasites in vitro [29], [30], and also prevent relapsing malaria [9], [31]. Unfortunately, its tendency to cause hemolytic anemia in patients with glucose-6-phosphate dehydrogenase deficiency, the most common human enzyme deficiency, has severely restricted its use (review in [32]). This deficiency is most common in certain parts of Africa (review in [32]), thus eliminating any hope of using primaquine in an eradication campaign. However, while primaquine itself can never be a widely used anti-malarial agent, its existence argues that other molecules with better therapeutic potential probably exist [12].

**Figure 2:**
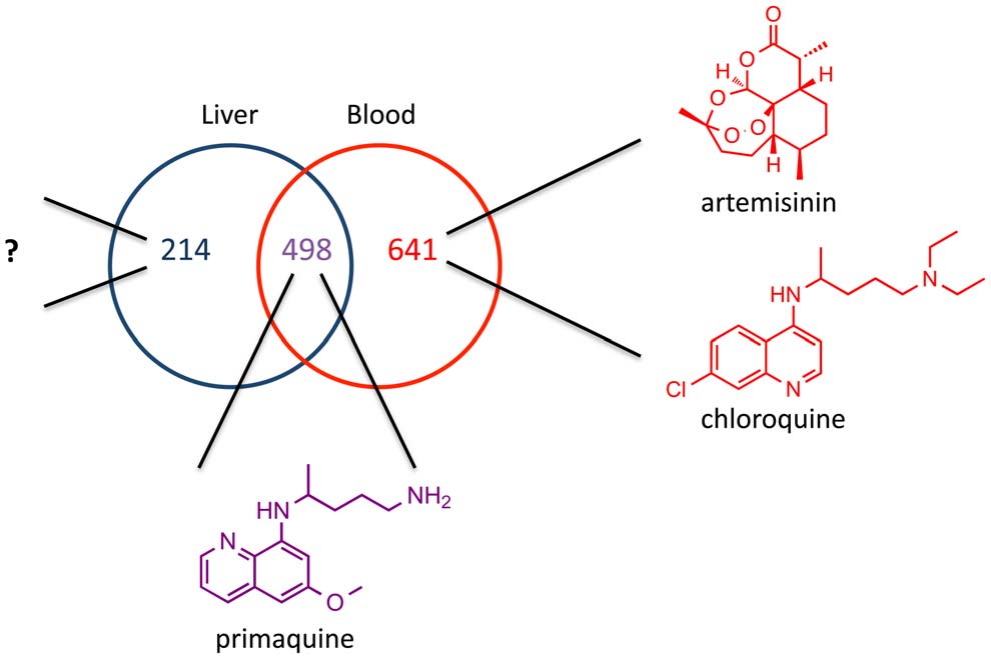
Potential for liver stage specific inhibitors. Venn diagram of overlap between *P. yoelii* liver stage schizont proteins [52] and *P. berghei* mixed blood stage proteins [53]. Analysis of *Plasmodium* proteomes was done by Tarun et al. Malaria drugs known to target the various stages are shown. Currently there is no drug on the market that selectively inhibits liver stage parasites.

Additional evidence for the likelihood of such drugs comes from transcriptional profiling of blood and liver stage parasites. Several recent reports have analyzed both transcriptome and proteome expression levels of malaria parasites in different life stages. These studies uniformly revealed a remarkable number of genes and proteins that are expressed *only* during the liver stage and thus represent likely stage-specific drug targets (see Figure 2) [33]–[35]. This marked difference between the two stages is unsurprising as the different parasite forms not only find, bind to, and efficiently invade different host cells, but also their replication rates differ by three orders of magnitude. Interestingly, the majority of the differentially expressed liver stage genes encode hypothetical proteins of unknown function [35], a finding that also suggests that they could reveal many new targets. A phenotypic liver stage screen would provide the most efficient path to discovering new drugs that would hit these targets.

## Prospects for a High-Throughput Phenotypic Screen for the Liver Stage

Our understanding of liver stage malaria research has advanced considerably in recent years, and the pieces for a high-throughput screen for liver stage infection appear to be in place. Arguably the most important advance was developing an in vitro culture system for liver stage infection. Now both human hepatoma cell lines and primary hepatocytes can be infected with sporozoites from different *Plasmodium* species isolated from mosquitoes (review in [36]), although the efficiency is still low. Infection monitoring was initially done with antibody imaging, which was very time consuming [37]–[39]. Still other methods including RT-PCR [40], [41] and fluorescence-activated cell sorting (FACS) using GFP-expressing parasites [42], [43] have emerged, and they allow both overall parasite load and *Plasmodium* development to be evaluated [44]. *Plasmodium* sporozoite infection and quantification of liver cells has even been accomplished in 96-well format [45]–[47], suggesting low to medium chemical screening is possible. Luciferase transgenic parasites have been reported in rodent *Plasmodium* strains (*P. yoelii* and *P. berghei*) [48], [49] and these bioluminescent parasites reduce the time of analysis and provide an efficient method to quantify parasite load both in vitro and in vivo.

With both in vitro culturing and reporting methods coming together for a quantitative high-throughput liver stage screen, we anticipate a shift from blood stage to liver stage for high-throughput phenotypic drug screens. It is likely that the rodent malaria strains, including *P. yoelii* and *P. berghei*, will be the first to be evaluated as the essential tools are already at hand. Of course, running an assay with today's tools requires the continual infection of liver cells with sporozoites and dissection of infected mosquitoes to isolate these sporozoites. Sporozoite harvesting would currently limit any high-throughput assay. In addition to being tedious, parasite harvesting has an inherent variability as it is difficult to standardize mosquito infections over long periods of time [12]. Still, cryopreservation of large batches of *Plasmodium* sporozoites may in the future overcome this limitation [50].

Unfortunately, less progress has been made towards reliable in vitro models for hypnozoite assays. To date the only widely used model to screen for anti-hypnozoite activity is via the infection of rhesus monkeys with *P. cynomolgi* sporozoites (reviewed in [9]). Recently slow growing *P. cynomolgi* hepatic forms were characterized after sporozoite infection of *Macaca fascicularis* primary hepatocytes [51]. Similarly, small parasite forms that may be hypnozoites have also been reported after infection of hepatoma cells with purified, cyropreserved *P. vivax* sporozoites [50]. These reports represent an important first step to establishing in vitro hypnozoite models, but further validation of the system is needed before screening efforts can begin. In particular, re-activation of such putative hypnozoites is required prior to any further development. In that context, in vitro infection of primary hepatocytes or hepatoma cells for longer periods of time must be optimized.

In summary, a high-throughput screen against liver stage parasites should be an attainable goal, although there are still challenges to overcome for truly high throughput. The urgent need for better anti-malaria strategies and the eventual eradication of malaria coupled with the remarkable recent progress from several laboratories make us optimistic about achieving a new generation of liver stage inhibitors in the near future.

Gene IDs for mentioned proteins are 2539 (glucose-6-phosphate dehydrogenase), 812272 (histone deacetylase), 9221804 (dihydrofolate reductase), 811999 (heat shock protein 90), and 3885966 (dihydroorotate dehydrogenase).
